# Mechanism of cellular uptake of genotoxic silica nanoparticles

**DOI:** 10.1186/1743-8977-9-29

**Published:** 2012-07-23

**Authors:** Qingshan Mu, Nicole S Hondow, Łukasz Krzemiński, Andy P Brown, Lars JC Jeuken, Michael N Routledge

**Affiliations:** 1 Centre for Molecular NanoScience (CMNS), University of Leeds, Leeds LS2 9JT, UK; 2 Institute for Membrane and Systems Biology, University of Leeds, Leeds LS2 9JT, UK; 3 Leeds Institute of Genetics, Health and Therapeutics, School of Medicine, University of Leeds, Leeds LS2 9JT, UK; 4 Institute for Materials Research, University of Leeds, Leeds LS2 9JT, UK

**Keywords:** Nanoparticles, Silica, Genotoxicity, Electron microscopy, Model membrane, Non-endocytotic uptake

## Abstract

Mechanisms for cellular uptake of nanoparticles have important implications for nanoparticulate drug delivery and toxicity. We have explored the mechanism of uptake of amorphous silica nanoparticles of 14 nm diameter, which agglomerate in culture medium to hydrodynamic diameters around 500 nm. In HT29, HaCat and A549 cells, cytotoxicity was observed at nanoparticle concentrations ≥ 1 μg/ml, but DNA damage was evident at 0.1 μg/ml and above. Transmission electron microscopy (TEM) combined with energy-dispersive X-ray spectroscopy confirmed entry of the silica particles into A549 cells exposed to 10 μg/ml of nanoparticles. The particles were observed in the cytoplasm but not within membrane bound vesicles or in the nucleus. TEM of cells exposed to nanoparticles at 4°C for 30 minutes showed particles enter cells when activity is low, suggesting a passive mode of entry. Plasma lipid membrane models identified physical interactions between the membrane and the silica NPs. Quartz crystal microbalance experiments on tethered bilayer lipid membrane systems show that the nanoparticles strongly bind to lipid membranes, forming an adherent monolayer on the membrane. Leakage assays on large unilamellar vesicles (400 nm diameter) indicate that binding of the silica NPs transiently disrupts the vesicles which rapidly self-seal. We suggest that an adhesive interaction between silica nanoparticles and lipid membranes could cause passive cellular uptake of the particles.

## Background

The unique physicochemical properties of nanoparticles (NPs) that have given rise to applications in many fields, including drug delivery [1], cancer therapy [2], biosensors [3], food additives and cosmetics [4], may also increase the risk of toxicity to humans or the environment [5]. Many *in vitro* studies have demonstrated that certain NPs are cytotoxic and can cause oxidative stress and DNA damage, which has raised human health concerns [5-10]. As more NPs and NP-containing products are developed and brought into commercial use, it is generally assumed that NPs will enter the environment [11]. Industrial production of NPs is increasing in scale and diversity, raising additional concerns of environmental exposure to nanomaterials. The potential for human and ecological toxicity associated with nanomaterials is thus a growing area of investigation [12]. The toxic effects of a wide range of sizes of silica NPs have been tested and despite the NPs showing fast agglomeration upon contact with cell culture media, smaller sized silica NPs have been shown to be more cytotoxic than larger ones, [13,14].

It is critical to understand fundamental mechanisms underlying any biological responses to NPs, be they desirable or not. Understanding the principles of how NPs can transmigrate into cells could enable greater control over cellular uptake and would improve prediction of possible toxic effects. There have been reports that some NPs are taken up by cells via non-endocytic pathways [11,15,16], and model membranes have indicated possible mechanisms for non-endocytic uptake [17-19]. For instance, Banerji *et al.* showed that citrate-capped gold particles (7 to 15 nm diameter) do not diffuse through a lipid membrane and can be encapsulated by vesicles [18]. In contrast, hydrophobic alkane-thiol coated gold NPs of 2 nm diameter become located inside the hydrophobic core of the lipid bilayer (whereas larger hydrophobic particles tend to disrupt lipid vesicles) [19]. A range of behaviours has been observed with amorphous silica particles. Particles up to 5 μm diameter have been shown to enter the cytoplasm of cells and although they are considered to have good biocompatibility, they have been shown to have haemolytic activity (see [20] and references therein). Mesoporous silica NPs cause haemolysis of mammalian red blood cells through interaction between the surface of the NPs with the cell membrane [20-22]. Silica NPs of less than 100 nm can induce endocytosis-dependent reactive oxygen species generation, DNA damage [23] and aberrant nucleoplasmic protein aggregation [24,25]. Furthermore, amorphous silica particles of 15–20 nm can bind to lipid vesicles, while larger particles (up to 190 nm) can transmigrate into giant unilamellar vesicles (GUV) [17]. In the latter case the silica particles were coated with a lipid membrane in the process. Membrane disruption by amorphous silica nanoparticles has also been identified by electrophysiological methods [26]. If such disruption were to enable uptake directly into the cytoplasm, without significant damage to the cell, silica nanoparticles would be potential vehicles for drug delivery applications. The biocompatibility of amorphous silica, its amenability to surface modification and the fact that it is not electro-active in aqueous media has already led others to consider it for application in gene and drug delivery [27,28].

The affinity of amorphous silica for lipid bilayers and the potential for ‘passive’ uptake in vesicles makes silica NPs an intriguing target for toxicological as well as drug-delivery studies. The objectives of this study were to explore the mechanism by which amorphous silica NPs (of 14 nm diameter) enter the cell and to evaluate *in vitro* cytotoxicity and the potential to induce DNA damage of the NPs. Dynamic Light Scattering (DLS) was used to identify agglomeration in the cell culture medium and transmission electron microscopy (TEM) was used to identify cellular uptake of the silica NPs. Biomembrane models were used to study the interaction with lipid membranes, to establish whether the silica NPs can induce structural changes to a phospholipid bilayer, thereby compromising the barrier function of the plasma membrane and inducing uptake.

## Results

### Dispersion in cell culture medium

**Figure 1:**
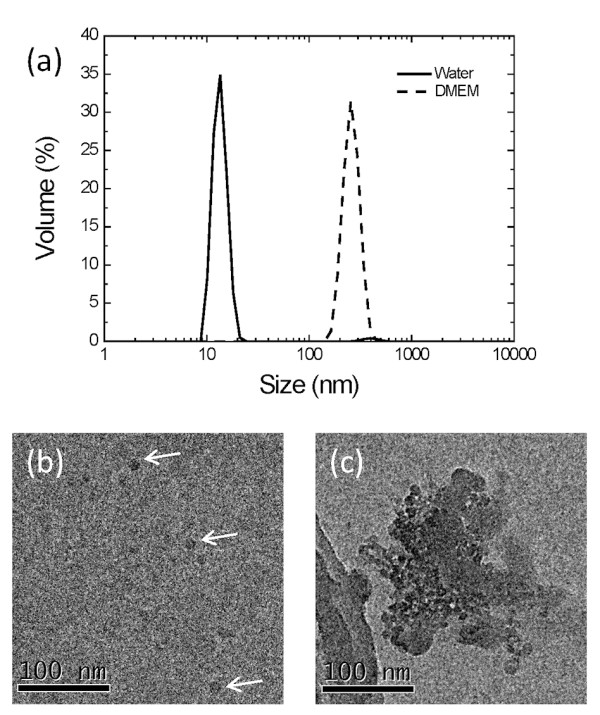
**(a) Dynamic light scattering measurements of silica NP (100 μg/ml) size distributions in MilliQ water (solid line) and DMEM (dashed line) at 37°C.** (**b**,**c**) TEM images of silica NPs (100 μg/ml) suspended in (b) Milli-Q water and (c) DMEM. Due to the relatively low electron density of SiO_2_ the TEM images of the dispersed silica NPs have low contrast (b) so for clarity, three isolated NPs are identified with arrows.

The size distribution of silica NPs at 37°C was determined by dynamic light scattering. In ultra-pure water (MilliQ, 18 MΩ·cm), the NPs were monodispersed with a dominant volume fraction around 14 nm (Figure 1a) and this was confirmed by TEM (Figure 1b). Suspended in Dulbecco’s Modified Eagle’s Medium (DMEM), however, the NP dispersion showed a dominant volume fraction at about 500 nm (Figure 1a), indicating that the particles significantly agglomerate or aggregate in this growth medium. Similar agglomeration was also observed in MOPS buffer (20 mM MOPS, pH 7.4, 30 mM Na_2_SO_4_) and phosphate-buffered saline (PBS) solution, indicating a general effect due to the ionic strength of the medium. The tendency to aggregate or agglomerate in DMEM was also confirmed by TEM (Figure 1c).

### Cyto- and genotoxicity

**Figure 2:**
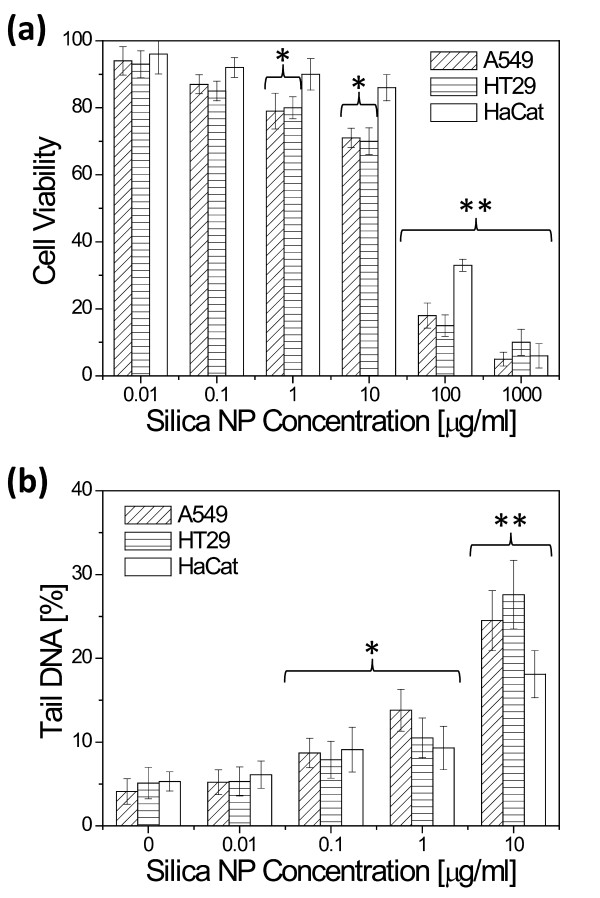
**(a) Viability of A549, HaCat and HT29 cells incubated with silica NPs for 24 hrs as determined by the MTT assay.** * and ** indicate that the viability is significantly lower than the control with p < 0.05 and p < 0.01, respectively. (**b**) DNA strand breaks induced by silica NP incubation for 24 hrs as determined by the Comet assay. * and ** indicate that the DNA tail is significantly longer than the control with p < 0.05 and p < 0.01, respectively.

Cytotoxicity was tested on three epithelial cell lines that were treated with varying doses of silica NPs for 24 hours. Cell viability determined by the MTT assay reduced significantly at a dose of 100 μg/ml and above (Figure 2a). In most cases, the HaCat cells exhibited increased resistance towards the silica NPs, compared to the other two cell lines.

Induction of DNA damage was determined using the Comet assay, also called single cell gel electrophoresis, which determines a combination of single-strand breaks and alkaline labile sites in individual cells [29]. Based on the cytotoxicity results, the three cell lines were treated with silica NPs at concentrations up to 10 μg/ml (significant cell death at higher concentrations hampers the interpretation of the assay results). The results (Figure 2b) show a significant increase in DNA damage compared to controls at concentrations of 1 and 10 μg/ml. Consistent with the results of the cytotoxicity assay, the HaCat cell line was the most resistant to DNA damage when incubated with 10 μg/ml silica.

## TEM results

**Figure 3:**
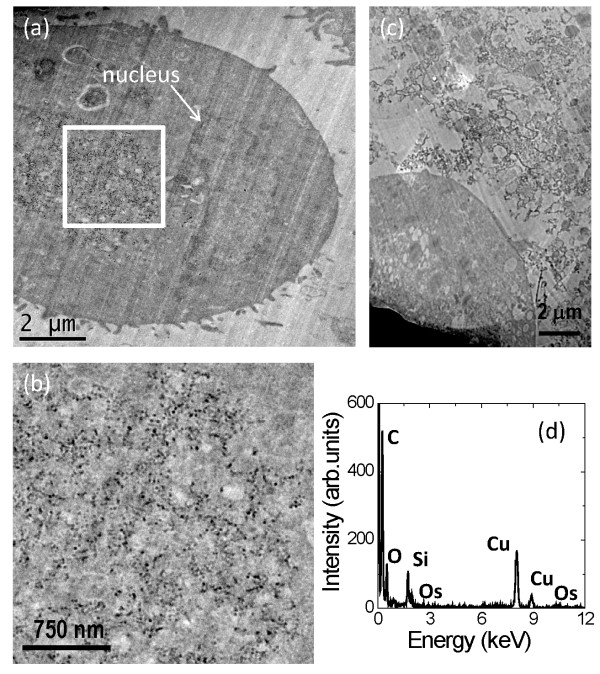
**(a) TEM image of an A549 cell after incubation with 10 μg/ml silica NPs for 24 hrs at 37°C.** (**b**) Higher magnification image of the area indicated by the box in (a). (**c**) TEM image of A549 cells after incubation with 100 μg/ml silica NPs for 24 hrs at 37°C, showing a corner of one of the few remaining cells and surrounding it a large amount of cell debris, which was predominant throughout the sections. (**d**) EDX spectrum from the silica NP containing region of the A549 cell shown in (b).

In order to determine whether the silica NPs are taken up by the cells, and if they are, where the NPs localise within the cell, TEM images were recorded for A549 cells incubated with 10 μg/ml and 100 μg/ml silica for 24 hours under identical conditions as the MTT and Comet assays. TEM of the cells incubated with 10 μg/ml silica showed that the majority of cells were intact and that silica NPs were present in the cytoplasm (Figure 3a, 3b). There was not much cell debris observed by TEM at this concentration. Due to the small size of the NPs and the low electron density of SiO_2_, energy-dispersive X-ray spectroscopy (EDX) was required to confirm that the particles in Figure 3b were indeed silica (Figure 3d). The particles were well dispersed or only loosely agglomerated in parts of the cell. Since conventional heavy metal staining masks the location of the NPs and they could only be detected in unstained sections, the precise cellular localisation of the particles was not easy to determine. However, the nucleus can be identified in the unstained sections (because the osmium tetroxide fixative lightly stains lipids) and the images consistently indicated that the silica NPs were not present in the nucleus (Figure 3a). Furthermore, we found no evidence that the particles were clustered in endosomes. Further TEM images are available in Additional files 1, 2, 3, 4, 5 and 6.

To confirm that the apparent location of the silica particles was not due to an artefact of the sectioning of the resin-embedded cell samples, specimen tilt series were recorded for Figure 3a and 3b (Additional files 7 and 8, respectively). Analysis of the tilt series confirmed that the silica NPs were located in the TEM section (rather than on top of the section) and were in the cytoplasm with no apparent membrane encapsulation.

Finally, from the TEM images it was also obvious that a higher silica dosage (100 μg/ml) caused significant cell death. Figure 3c shows part of one of the very few intact cells that were observed in the TEM sections and the large amount of cell debris around the cell, possibly mixed with the NPs. The low number of intact cells is consistent with the cytotoxicity results.

**Figure 4:**
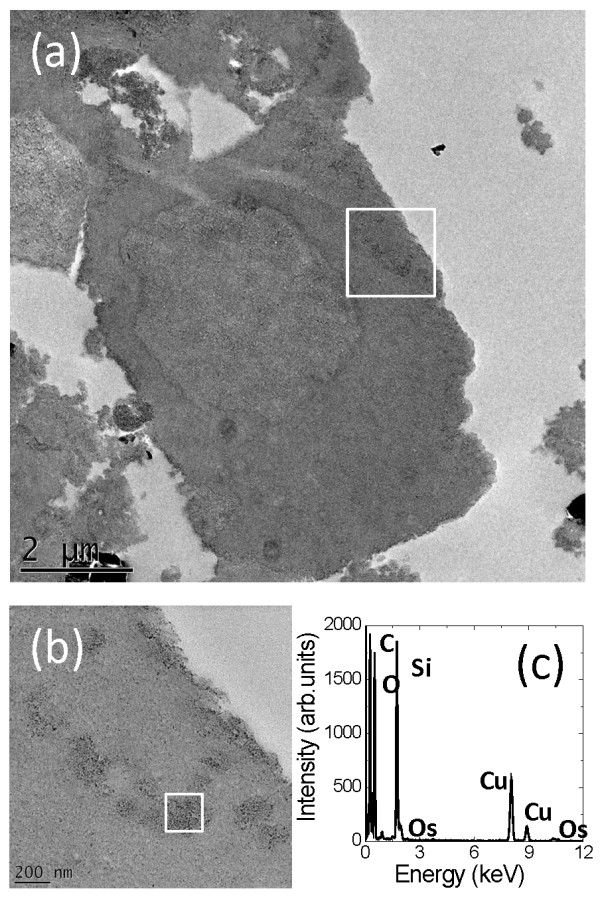
**(a) TEM image of A549 cellular uptake of silica NPs after incubation with 100 μg/ml for 30 mins at 4°C.** (**b**) Higher magnification image of the area indicated by the box in (a). (**c**) EDX spectrum from the silica NP containing region of the A549 cell indicated by the box in (b).

Internalization of NPs into the cell types tested here usually takes place by endocytosis [30-32], but some reports have emerged in which non-endocytic pathways are proposed [11,15,16]. To explore cellular uptake mechanisms of silica NPs by A549 cells, particle exposure was carried out for 30 min incubation at 37°C and 4°C. TEM-EDX was again used to confirm the presence of silica NPs inside the cells (Figure 4 and Additional files 3, 4, 5 and 6). The cells incubated with 100 μg/ml silica NPs at 37°C for 30 min showed a similar uptake of NPs as those incubated with 10 μg/ml silica NPs at the same temperature for 24 hours. Importantly, the cells incubated at 4°C, at which temperature active cell processes are significantly suppressed, also showed NP uptake and, again, the NPs themselves were spread out in the cytoplasm without obvious membrane encapsulation (Figure 4). Furthermore, at 4°C the cellular membrane seems to be densely covered with NPs, although there is no evidence of structural damage to the membranes. The ‘wavy’ structure of the cell edge was also found in the control cells incubated at 4°C and is thus likely to be a temperature effect (Additional file 4).

### Model membranes

**Figure 5:**
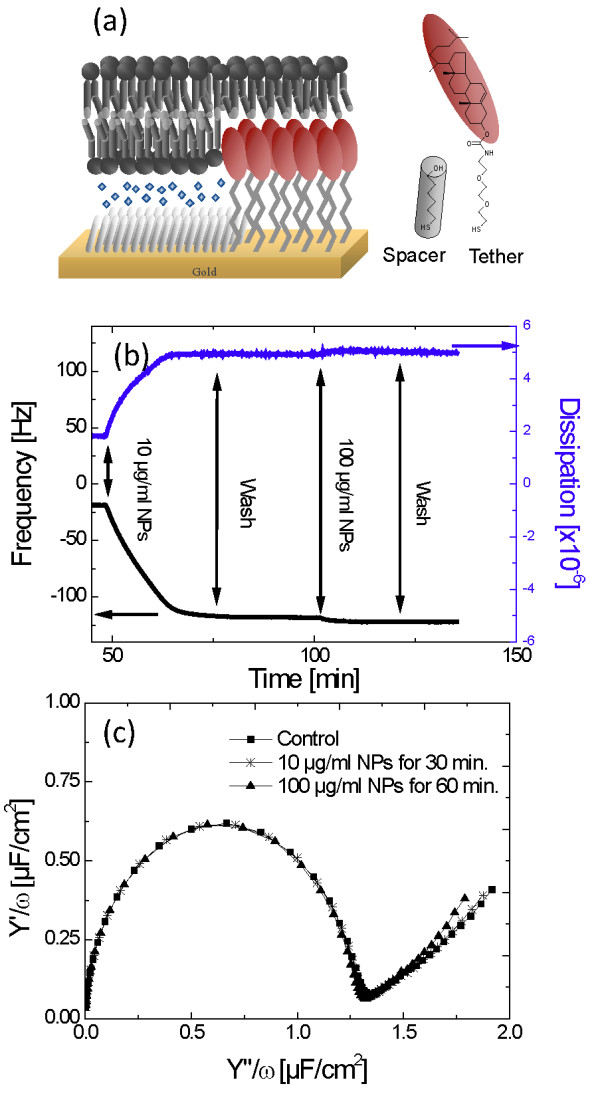
**(a) Schematic of the tBLM system used in (b) and (c).** A gold surface is modified with a mixed self-assembled monolayer containing cholesterol groups. The surface is then incubated with vesicles made up of Lipid Mix 1 (POPC:DOPS:cholesterol at 15:1:4 weight ratio), which assembles into a planar membrane across the gold surface, (b) QCM-D plots of a tBLM in DMEM after additions of 10 and 100 μg/ml of silica NPs (dispersed in DMEM) and after washes with DMEM at times indicated by the arrows. (c) Impedance spectra plots (normalised admittance) of a tBLM in DMEM before and after incubation with 10 and 100 μg/ml of silica NPs at 21°C for the indicated time.

At concentrations of NPs ≥ 100 μg/ml, MTT assays and TEM indicated that the cells are not viable and undergo lysis. Below 100 μg/ml, TEM analysis leads to the suggestion that the silica NPs were taken up via a non-endocytic pathway. In order to test if both effects could be due to a strong interaction between the particles and the plasma membrane, resulting in the physical breakdown of the membrane, the interaction of the NPs with various model membranes was studied. The binding of NPs and breakdown of model membranes was studied using a tethered bilayer lipid membrane (tBLM) system. In the tBLM system, a lipid bilayer is attached to a metal electrode in a planar orientation (Figure 5a).

The formation of the lipid bilayer in the tBLM system and the binding of particles to the lipid membrane can be monitored by Quartz-Crystal Microbalance with Dissipation (QCM-D). In Figure 5b, the formation of the tBLM is not shown for clarity. The oscillation frequency of around -20 Hz at the beginning of the trace is due to the mass of the lipids in the tBLM. Upon addition of 10 μg/ml silica NPs, a very strong decrease in frequency was observed indicating that the NPs bind to the surface of the membrane. At the same time, the dissipation of the oscillation only rose by around three units, indicative of a tight interaction between rigid NPs and the lipid membrane. This is further supported by the fact that rinsing the system did not change the frequency or dissipation, suggesting that rinsing does not release the NPs from the membrane (Figure 5b). A further addition of 100 μg/ml did not induce a large change in the QCM-D response, indicating that the lipid membrane was already saturated with NPs. Using the Sauerbrey equation, it is calculated that 2.1 μg/cm^2^ silica NPs bind onto the tBLM layer after addition of 100 μg/ml in solution. A fully-packed hexagonal arrangement of a monolayer of 14-nm silica NPs (density 2.648 g/cm^3^) has an areal mass of 2.24 μg/cm^2^. When the data is analysed using a Voigt model that assumes two layers (a fixed layer for the tBLM and a second layer for the silica), a silica layer thickness of 17.6 nm is obtained, again consistent with a well packed monolayer of 14-nm silica NPs on the lipid membrane. This dispersion across the membrane indicates that the silica NP agglomeration, which occurs upon dispersion in DMEM (Figure 1), can be broken down and as such is not aggregation.

**Figure 6:**
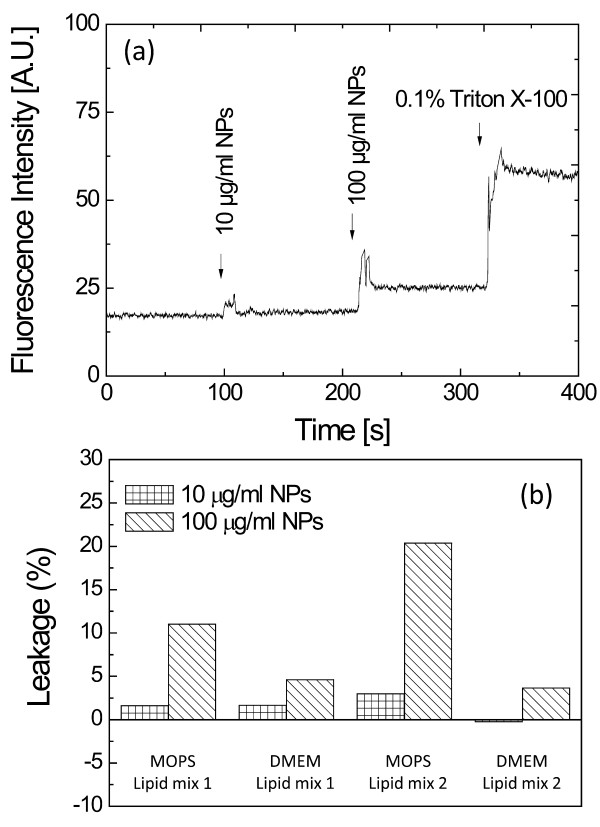
**(a) Fluorescence intensity of carboxyfluorescein-loaded vesicles in 20 mM MOPS, 30 mM Na**_**2**_**SO**_**4**_**, pH 7.4 after the addition of 10 and 100 μg/ml of silica NPs and 0.1% (v/v) Triton X-100, at times indicated by the arrows.** The vesicles are made up of Lipid mix 1 (POPC:DOPE:DOPS:cholesterol at 9:6:1:4 weight ratio) and loaded with 50 mM carbofluorescein. (**b**) Leakage of carboxyfluorescein from 400 nm vesicles after the addition of silica NPs under various conditions, as indicated (MOPS = 20 mM MOPS, 30 mM Na_2_SO_4_, pH 7.4; DMEM = Dulbecco’s Modified Eagle’s medium; Lipid Mix 1 = POPC:DOPS:cholesterol at15:1:4 weight ratio; Lipid Mix 2 = POPC:DOPE:DOPS:cholesterol at 9:6:1:4 weight ratio). Leakage (as a percentage) was determined assuming 0% leakage prior to addition of NPs and 100% leakage after the addition of 0.1% (v/v) Triton X-100 as shown in (a).

It is possible that the tightly-bound NPs physically damage the plasma membrane, potentially permeabilising it to polar compounds and NPs. To study this proposition, the tBLM was characterised using electrochemical impedance spectroscopy (EIS), which is sensitive to ion transport through a membrane. The tBLM is unperturbed by the addition of NPs up to 100 μg/ml (Figure 5c), suggesting that the plasma membrane remains a constructive barrier upon binding of silica NPs. We note, however, that EIS is insensitive to rare, dynamic perturbations of the membrane. To study whether silica NPs can enter cells by *transient* membrane disruption, vesicle-leakage assays were performed. 400-nm diameter vesicles of various lipid compositions were loaded with an auto-quenching fluorescent dye and incubated with the NPs. Release of the dye from a vesicle results in a dilute concentration in solution and thus raises the observed fluorescence intensity. Indeed, significant fluorescence increases were observed upon addition of NPs, indicating some dye is released and the lipid membrane is compromised (Figure 6a). The real time fluorescence data is quantified in Figure 6b. Up to 20% of the dye was observed to be released by the vesicles upon addition of 100 μg/ml NPs. It is also noticeable that more significant effects were found in MOPS buffer than in DMEM (Figure 6b). We note that the lipid content in fluorescent leakage assays was 15 μg/ml and that the number of NPs far exceeded the number of vesicles in solution. At 100 μg/ml, enough silica NPs were present to fully cover the lipid vesicles, but at 10 μg/ml NPs it is estimated that only a submonolayer can be formed.

## Discussion

It is recognised that the potential dose of silica particles depends on mass concentration, particle number or surface area. It has been noted previously that silica NPs show fast agglomeration and some dissolution upon contact with cell culture media, which directly influences the uptake into the cell [13,21]. We have used well characterised amorphous silica NPs of 14 nm diameter to explore the interaction of sub-lethal concentrations of NPs with cultured A549, HT29 and HaCat cells. We found that the particles aggregated or agglomerated in DMEM culture medium to form assemblies of NPs around 500 nm (Figure 1). Previous results have indicated that larger particles could cause greater disruption of the membrane than smaller particles [20,33]. Here, nearly all cells lysed after 24 h exposure to a NP concentration of 100 μg/ml (Figure 2), consistent with previous findings for amorphous silica NPs below 100 nm diameter exposed for A549 cells [34] and several other cell types [13,14,23,35-37] exposed to amorphous silica NPs below 100 nm diamter.

Silica NPs have been shown to induce caspase activation and cell death by apoptosis, however, the pathway leading to apoptosis is controversial [38-40]. It has been suggested by Wittmaack [41,42] that *in vitro* cytotoxicity due to silica and other NPs is strongly influenced by gravitational settling of the particles forming high concentrations on top of the cells in the cultures and it is this ‘coating’ which then interferes with membrane mediated processes. In our experiments, TEM of cells exposed for 24 h to 100 μg/ml silica NPs showed that nearly all cells had already lysed, with large clumps of silica particles visible in the debris (Figure 3c).

Previous studies do not report a consistent threshold dose of amorphous silica NPs above which genotoxicity is detectable (see ref. [13] for an extensive review on silica toxicity). Up to 40 μg/ml silica NPs did not induce genotoxicity in fibroblast cells in a comparative study in which three laboratories carried out the Comet assay [43]. In contrast, in this study, significant DNA damage was observed in the Comet assay in three different cell lines at only 10 μg/ml, at which dose silica particles were found inside the cytoplasm of A549 cells, but not in the nucleus (Figures 2 and 3). In our study the NP dosing for the Comet assay was undertaken in serum-free DMEM whereas Barnes et al. dosed in DMEM supplemented with 10% foetal bovine serum (FBS) [43]. Serum proteins have been shown to interact with silica NPs [36] with serum concentration reducing the amount of cellular uptake of iron oxide nanoparticles and therefore the biological impact of exposure, particularly where genotoxicity assays are concerned [44].

It has previously been reported that reactive oxygen species (ROS) are responsible for toxic effects from crystalline silica NPs [13,45] and that ROS are induced in cells that have taken up amorphous silica, leading to cytotoxicity [46] and genotoxicity [23]. The absence of particles in the nucleus suggests that the DNA damage is due to a cellular response to the presence of particles inside the cell. Here the particles inside the cytoplasm do not appear to be associated with or encapsulated by internal membrane systems although with the current TEM specimen preparation of only 1% osmium tetroxide fixative and no additional heavy metal stains it is difficult to prove this (Figures 3 and 4). However, endocytic uptake of iron oxide NPs and carbon nanotubes has been readily identified for other cells types that have been prepared for TEM in a similar manner [47,48]. More importantly, non-membrane bound uptake of amorphous silica NPs (43 nm diameter) has previously been identified by TEM, alongside uptake in endocytic vesicles, in a hepatocellular carcinoma (HepG2) cell line exposed in serum-free culture media [49]. This contrasts with most previous studies that have shown silica NPs delivered in cell culture media supplemented with serum proteins, tend to be taken up by endocytosis and, in general, to be associated with internal membranes once inside the cell [50,51]. The TEM results here suggest that a non-endocytic pathway is in operation in addition to the expected (endocytic) routes. The experiments performed at 4°C indicate that active cellular processes are not required for uptake of the particles into the cells (Figure 4). Uptake of silica NPs in red blood cells (RBC), which do not carry out active endocytotic uptake, has already been demonstrated [20-22] and recently it has been shown that zwitterionic quantum dots can passively cross the cell membrane of erythrocytes [52].

In addition, amorphous silica particles can cross lipid bilayers and enter giant unilamellar vesicles (GUV), although particles smaller than 20 nm apparently only bind to and do not enter the lipid vesicles [17]. The silica NPs in our study form 500 nm agglomerates in the cell culture medium (Figure 1) and our QCM-D studies suggest that these agglomerates can disperse on contact with the membrane to form a densely packed silica monolayer coating (Figure 5b). The electrochemical impedance spectroscopy, however, is not affected by the addition of silica particles (up to a dose of 100 μg/ml), indicating that 14 nm silica particles are not able to structurally impair the lipid membrane (Figure 5c). This would suggest that these silica NPs only bind to lipid vesicles and cells without further penetration, which differs from previous results that showed disruption of membranes by bound silica nanospheres covering less than 1% of the surface of a lipid bilayer [26]. However, our dye leakage experiments clearly indicate that liposomes, composed of lipid mixtures typical of cellular plasma membranes, are transiently, structurally disrupted immediately after mixing with the silica NPs (Figure 6). Previous studies show that there is a size dependent response to RBC membrane disruption by silica NPs and so the agglomeration or aggregation of the silica NPs in our study might still be important [22]. A model could be proposed in which leakage is induced by the lipid membrane wrapping or bending around the silica agglomerates before they can disperse across the membrane. The reduced curvature around large agglomerates could be required to accommodate the limited bending capacity of the lipid membrane. This model is consistent with the absence of a response of the tethered bilayer system (Figure 5), which is hindered from bending because it is tethered to a planar surface.

An alternative model might operate where the silica NP agglomerates fully disperse across the vesicle membrane, (as per the tethered bilayer measurement; Figure 5) and the consequent coverage of vesicles induces a strain that is released by transient pore formation. Significantly, in our study only part of the dye is released upon addition of NPs and the disruption of the vesicles is only observed for a very short time after NP addition. This indicates that the membrane disruption upon the addition of silica NPs is transient and the resulting pores reseal rapidly, also consistent with the absence of effects in the impedance spectra (Figure 5). Considerable leakage is only observed at 100 μg/ml, while much less leakage is observed at 10 μg/ml (Figure 6). It is striking that only at 100 μg/ml there are sufficient numbers of silica NPs to fully coat the 400 nm lipid vesicles in this leakage assay, suggesting that a full monolayer of silica particles might indeed be required to induce leakage. Previous studies using bile salts have suggested that asymmetric binding to the lipid membrane of vesicles can induce strain, resulting in transient membrane pores which release this strain [53]. The disrupting effect of bile salts was dependent on the lipid composition as well as on chemical additions to the buffer (e.g. glycine). Here, an influence of both lipid and buffer composition is also observed. In fact, certain lipid compositions such as total sheep brain lipid extracts are unperturbed by silica NP in the dye-release assay (data not shown).

Recently, confocal microscopy studies were performed on DOPC giant unilamellar vesicles (GUVs) exposed to 1–100 μg/ml of silica NPs at physiological pH for 20 minutes, which confirm the formation of stable, micrometre-size pores and unusual crinkles in the GUVs [54]. The formation of pores and crinkles has been attributed to the significant reduction in lateral lipid mobility and lipid free volume after the adsorption of NPs onto the lipid membrane. We hypothesise that a similar mechanism operates at the plasma membrane of the cell*;* doses of 10 μg/ml silica NPs induce the formation of transient pores to allow uptake of the NPs into the cells. The exact mechanism of cell death at higher doses could be due to (a) increased amounts of NP uptake and the cell’s response to the particles in the cytoplasm, (b) increased amounts of transient pores breaking down the plasma membrane or (c) effects that silica NPs exert on the plasma membrane upon binding, such as changing its fluidity or impairing membrane protein function.

## Conclusion

We show that for a range of cell types, amorphous silica NPs, 14 nm in diameter but agglomerated to 500 nm in cell culture medium, cause reduced cell viability and can induce DNA damage. The precise impact of serum proteins in the cell growth media on particle agglomeration, uptake and toxicity is still to be determined. We suggest that for A549 cells, exposure in the absence of serum proteins results in silica NP uptake to the cytoplasm directly. Tethered model membrane experiments indicate that the NPs bind to membranes to form a densely-packed monolayer without significant membrane disruption, while for unilamellar vesicles the NPs induce transient membrane disruption. This adhesive interaction with lipid membranes suggests amorphous silica NPs can be passively transported into cells. These data also indicate that silica NPs are unlikely to have significant human health effects at environmental exposure levels, although the ecotoxicology of such particles has still to be determined. The particles might thus be a suitable vehicle for drug delivery and gene therapy.

## Methods

### Materials and NP characterisation

All chemicals were purchased from Sigma (UK) unless stated otherwise. Mercapto-ethyleneoxy_3_-cholesterol (EO_3_C) was synthesised as previously described [55].

The amorphous silica NPs (Ludox SM-30) used in this study were purchased from Ludox Colloid Silica and dialysed for three days against MilliQ water (18 MΩ cm). Nanoparticle dispersions were diluted in MilliQ water and DMEM at 100 μg/ml for subsequent characterisation after leaving the dispersions to stabilise for at least 24 hours. ICP-MS (Perkin Elmer Elan DRC-e) of a 10 μg/ml silica sample in MilliQ water did not detect any transition metals in the sample. Dynamic light scattering (DLS) of NP dispersions were conducted at 37°C using a Malvern Zetasizer Nano ZS and the Zetasizer software (Version 6.20). Each dispersion was analysed 3 times with the average result presented. Transmission electron microscopy (TEM) of the NP dispersions was conducted on an FEI Tecnai F20 FEG-TEM operating at 200 kV equipped with a Gatan Orius SC600A CCD camera. TEM samples were prepared by plunge-freezing: a 3.5 μL droplet of each dispersion was placed on a glow discharge treated carbon support film (R1.2/1.3 Quantifoil MicroTools GmBH), blotted and plunge frozen in liquid ethane [56]. The grids were then warmed under vacuum to devitrify and sublime the ice prior to imaging in the TEM.

### Cell culture

A549 (human lung alveolar carcinoma) cells and HT29 (human colon cancer) cells were cultured in DMEM containing 10% foetal bovine serum (Lonza, Slough, UK) with 0.5% penicillin streptomycin. HaCat (human keratinocyte) cells were cultured in RPMI media (Gibco, Paisley, Scotland) containing 10% foetal bovine serum (Lonza, Slough, UK) with 0.5% penicillin streptomycin. All cell lines were incubated at 37°C in humidified 5% CO_2_ until they were approaching confluence when they were harvested using 10% trypsin-ethylene diamine tetra-acetic acid (EDTA) and then re-seeded.

### MTT assay

Effects of silica NPs on the viability of A549, HT29 and HaCat cells were evaluated using the MTT assay (thiazolyl blue tetrazolium bromide). Cells were seeded in a 96 well plate (Fisher) at a density of 20,000 cells/well in DMEM with 10% fetal bovine serum and allowed to attach overnight at 37°C with 5% CO_2_. After removing the culture medium by two washes with phosphate buffered saline (PBS), the toxicity assay was started with 225 μL serum-free DMEM with specified amounts of silica (0–1000 μg/ml) for 24 hours. The MTT assay was performed according to the manufacturer’s protocol (Sigma). Optical absorbance was read using a Labsystems iEMS Reader MF at 540 nm. The results are expressed as percentage viability compared with untreated controls.

### Comet assay

A549, HT29 and HaCat cells were plated at a density of 120,000 cells/well in 24-well plates (Fisher) in medium overnight, after which they were incubated with silica NPs in serum free DMEM for 24 hrs at 37°C with 5% CO_2_. The cells were pelleted by centrifugation at 1000 rpm for 5 min and resuspended in serum-free DMEM to a concentration of 1x10^6^ cells/ml. A 100 μL aliquot of this suspension was mixed with 200 μL of 1% (w/v) low-melting-point agarose in phosphate buffered saline (PBS) solution and kept at 37°C until use. 100 μL was placed onto duplicate microscope slides (Thermo Scientific) pre-coated with 1% (w/v) low-melting-point agarose and covered with a coverslip (Scientific Laboratory Supplies Ltd.). Slides were placed on ice to allow the agarose to solidify, after which the coverslips were removed. The slides were treated with a detergent lysis solution (2.5 M NaCl, 1 mM EDTA, 10 mM Tris, 10% DMSO, 1% Triton X-100 at pH 10) for 1 hour and placed in running buffer (300 mM NaOH, 1 mM EDTA at pH 13) at 4°C for 40 min to allow DNA unwinding, followed by electrophoresis at a constant voltage of 23 V for 20 min. Slides were finally removed, neutralised by adding 400 mM Tris at pH 7.5 for 5 min, gently dried and stained with 30 μL ethidium bromide (25 μg/ml). Coverslips were placed onto each gel and the stained slides were stored in damp conditions at 4°C. The slides were viewed using an Olympus BX41 microscope and digitally analysed using Komet 5.5 software. Cells were scored by evaluating 50 cells per slide, with duplicate slides for every sample.

### TEM sample preparation

Cell culture sample preparation for TEM was identical to that used in the Comet assay. After incubation with silica NPs, cells were trypsinized, harvested by centrifugation at 1000 rpm for 5 min and kept on ice until use. The preparation of 4°C samples followed the same cell culture procedure as the 37°C samples except these were incubated with silica NPs at 4°C for 30 min. In both cases, the cell pellet was fixed with 2.5% glutaraldehyde in PBS for 2.5 hours, followed by two 30 min PBS washes. Osmium tetroxide, 1.0% (w/v), was added to the fixed cells and allowed to incubate for 16 hours. After another two 30 min PBS washes the cell sample was dehydrated by adding ethanol sequentially from 20% to 100% with 20% step increases for 30 min each time. In order to embed the cells, the sample was washed with propylene oxide twice for 20 min, then incubated in propylene oxide and araldite (1:1) for 16 hours, followed by transfer to propylene oxide and araldite (1:3) for several hours and finally transfer into pure araldite for 8 hours. The cells were transferred to embedding moulds containing fresh araldite, which was then polymerised for 16 hours at 60°C. The region of interest was cut off and glued onto a blank flat-end beam capsule using super glue and baked dry for 1 hour, before trimming and cutting ultrathin sections of 60 nm thickness with an ultramicrotome and diamond knife.

TEM was conducted on two microscopes; an FEI CM200 field emission gun (FEG-)TEM running at 197 kV equipped with an Oxford Instruments energy dispersive X-ray (EDX) spectrometer and a Gatan Imaging Filter (GIF-200) and an FEI Tecnai F20 FEG TEM operating at 200 kV fitted with a Gatan Orius SC600A camera and an Oxford Instruments energy dispersive X-ray (EDX) spectrometer. The tilt series was collected through a tilt range of +46 to −46 degrees with an image recorded every one degree on the Gatan Orius SC600A camera and processed using the software ImageJ [57], with the TomoJ plugin [58]. Images in the tilt series were defocused (underfocus) by 180 μm from minimum contrast to enhance the contrast of the amorphous NPs in the unstained cell section.

### Model membrane studies

The lipids 1-palmitoyl-2-oleoyl-sn-glycero-3-phosphocholine (POPC), 1,2-dioleoyl-sn-glycero-3-phospho-L-serine (sodium salt, DOPS), 1,2-dioleoyl-sn-glycero-3-phosphoethanolamine (DOPE) and cholesterol (ovine wool) were purchased from Avanti Polar Lipids (Alabaster, AL) (all lipids at purity > 99%). Two liposome mixtures were prepared: Lipid Mix 1 (POPC:DOPS:cholesterol at 15:1:4 weight ratio) and Lipid Mix 2 (POPC:DOPE:DOPS:cholesterol at 9:6:1:4 weight ratio). The lipid mixtures were prepared by mixing solutions of the pure lipids (dissolved in (1:1) methanol/chloroform), fractioned in 5 mg total lipid aliquots in glass vials and dried under vacuum for at least 2 hours. Aliquots of lipid mixtures were stored under nitrogen at −20°C until use. Vesicles were prepared by dispersing 5 mg/ml lipid in MOPS buffer (20 mM 3-(N-morpholino)propanesulfonic acid, 30 mM Na_2_SO_4_, pH 7.4) by vortexing and tip sonicating (Branson Sonicator 250) at 4°C for 25 min. Titanium particles from the tip were removed by spinning at 14,500 × g for 4 min.

Impedance spectroscopy of tethered bilayer lipid membranes (tBLMs): The preparation of the tBLMs xon template-stripped gold electrodes and the characterisation with electrochemical impedance spectroscopy (EIS) were performed in MOPS buffer, as described previously [59]. After replacing the MOPS buffer with DMEM solution, 10 μg/ml and/or 100 μg/ml silica NPs were added and the impedance monitored for the time indicated in the results section. All experiments were run at 20^o^C. Controls were recorded in the absence of silica NPs.

Quartz-crystal microbalance with dissipation (QCM-D): QCM-D experiments were performed on a QSense E4 (Gothenburg, Sweden). Gold coated quartz crystals were cleaned by sonicating them in 10% Decon in a water bath, rinsing with Milli-Q water, drying under a flow of nitrogen and then exposing them to a UV-Ozone cleaner (UVOCS Inc.) for 15 min and finally by incubating them in distilling ethanol (Soxhlet extractor) for another 30 min. tBLMs were formed on the gold-coated crystals in MOPS buffer, as described previously [59]. All QCM-D experiments were conducted under a flow rate of 100 μL/min at 21°C. Changes in the dissipation, *ΔD*, and normalized frequency, *Δf* (*f* = *f*_n_/n, where n is the number of the overtone, i.e., n = 3, 5, 7, etc.) of the ninth overtone (n = 9, 45 MHz) are presented in this work. Due to the mass of the lipids that form the tBLM on the QCM-D crystals, the traces in the result section start at about −20 Hz (the QCM-D responses to the formation of the tBLMs are not shown). After exchanging the buffer solution with DMEM, silica NPs (10 μg/ml) were introduced to the tBLM until no further changes in frequency or dissipation were observed, followed by a DMEM wash. Finally, silica at a concentration of 100 μg/ml was introduced. Control experiments were made without silica NPs. The data was analysed using the QSense software, Qtools.

Leakage assays: Carboxyfluorescein (CF)-encapsulated vesicles were prepared as described above for lipid vesicles, except that the lipid was resuspended in 1 ml of CF solution (50 mM) in MOPS buffer, vortexed and extruded through track-etched membranes (400 nm) using an Avanti extruder. Non-encapsulated CF was removed by size-exclusion chromatography with Sephadex G-25 (NAP-5 columns, GE Healthcare). Leakage of CF out of the vesicles as a function of time was monitored at 21°C by fluorescence with excitation at 492 nm and emission at 517 nm. The CF-loaded lipid vesicles were diluted at least a 100 times in either MOPS buffer or DMEM and equilibrated at room temperature for 3 hours. Fluorescence time scans were taken with a Perkin Elmer Lambda 35 spectrofluorophotometer. After monitoring the sample for 10 min, silica NPs were added at the indicated concentration and the fluorescence intensity recorded over time. A complete release of CF from the vesicles was achieved at the end of the experiment by adding 0.1% Triton X-100 to lyse the vesicles.

## Competing interests

The authors declare that they have no competing interests.

## Authors’ contributions

LJCJ, MNR, APB, and QM were involved in the design of the study. QM performed the MTT and Comet assay studies. QM, NSH and APB performed the TEM studies. QM and LK performed the model membrane studies. QM, LJCJ, APB and MNR contributed to the interpretation of data and the preparation of the manuscript. All authors approved the final manuscript.

## Supplementary Material

Additional file 1 **TEM bright field images of an A549 cell after 24 h incubation at 37°C with 10 μg/ml amorphous silica NPs (Figure **3** of main text).** Click here for file

Additional file 2 **Higher magnification of a boxed region from Additional file ****1****.** Click here for file

Additional file 3 **Further EM images of A549 cell after 30 min with 100 μg/ml silica NPs at 4°C shown in Figure **4** of the main text.** Click here for file

Additional file 4 Six figures showing TEM images of A549 cells after 30 min with and without 100 μg/ml silica NP exposures at 4°C and 37°C. Click here for file

Additional file 5 Additional EM images of A549 cells incubated at 4°C for 30 min with a 100 μg/ml of silica NPs. Click here for file

Additional file 6 Higher Magnification bright field TEM image and false colour elemental map of A549 cell incubated at 4°C for 30 min with a 100 μg/ml of silica nanoparticles. Click here for file

Additional file 7 **Tilt series of TEM bright field images of an A549 cell after 24 h incubation at 37°C with 10 μg/ml amorphous silica nanoparticles (Figure **3** of the main text).** This tilt series confirms that the silica particles are located within the cell. Click here for file

Additional file 8 **Tilt series of higher magnification of the boxed region in Additional file ****1****.** This tilt series confirms that the silica particles are located within the cytoplasm of the cell without membrane encapsulation and are not just on the surface of the ultra-thin section. Click here for file
