# Supplementary material for: Mechanism of cellular uptake of genotoxic silica nanoparticles
Source: Part Fibre Toxicol. 2012 Jul 23;9:29. doi: 10.1186/1743-8977-9-29 (PMC3479067; doi:10.1186/1743-8977-9-29)
Supplement: Additional file 1 — TEM bright field images of an A549 cell after 24 h incubation at 37°C with 10 μg/ml amorphous silica NPs (Figure 3 of main text). [file 1743-8977-9-29-S1.pdf]

Mu Q et al

Additional File 1

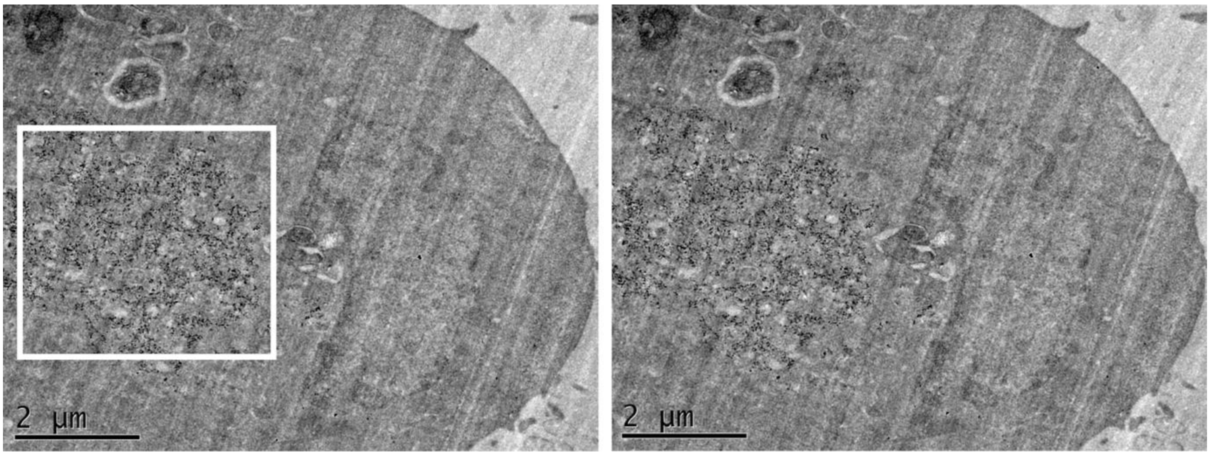

TEM bright field images of an A549 cell after 24 h incubation at 37°C with 10 µg/ml amorphous silica nanoparticles (Figure 3 of the main text). The images are a stereo pair (angular separation of 6 degrees) taken from a tilt series demonstrating that the silica nanoparticles identified in Figure 3 and boxed above are located in the sectioned cell. A movie of this tilt series is also available in additional file 7.
