# Supplementary material for: Mechanism of cellular uptake of genotoxic silica nanoparticles
Source: Part Fibre Toxicol. 2012 Jul 23;9:29. doi: 10.1186/1743-8977-9-29 (PMC3479067; doi:10.1186/1743-8977-9-29)
Supplement: Additional file 2 — Higher magnification of a boxed region from Additional file 1. [file 1743-8977-9-29-S2.pdf]

Mu Q et al

Additional File 2

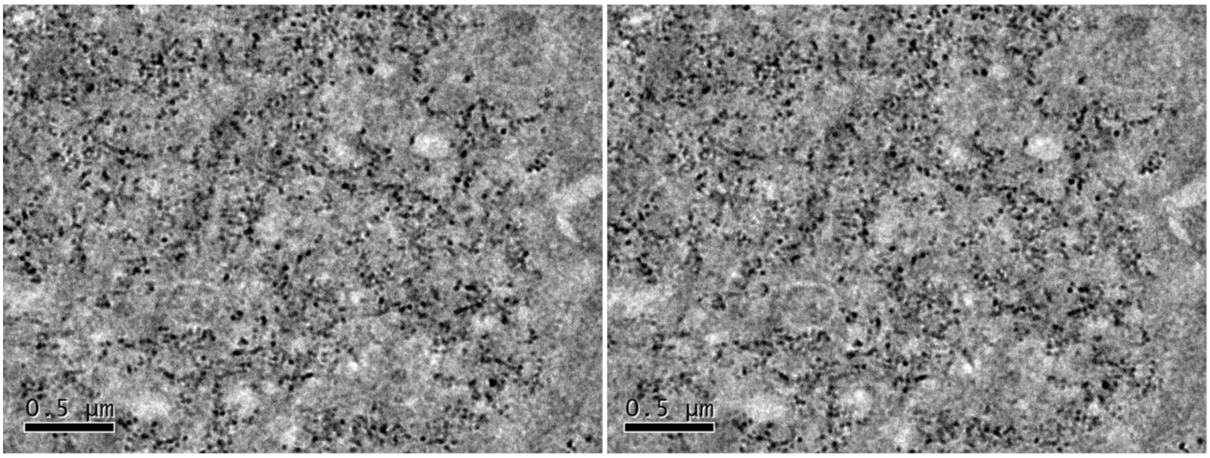

Higher magnification of the boxed region in additional file 1. This stereo pair of images (angular separation of 6 degrees) taken from a tilt series that confirms that the silica particles are located within the cell and are not just on the surface of the ultra-thin section. A movie of this tilt series is also available in additional file 8.
