# Supplementary material for: Mechanism of cellular uptake of genotoxic silica nanoparticles
Source: Part Fibre Toxicol. 2012 Jul 23;9:29. doi: 10.1186/1743-8977-9-29 (PMC3479067; doi:10.1186/1743-8977-9-29)
Supplement: Additional file 3 — Further EM images of A549 cell after 30 min with 100 μg/ml silica NPs at 4°C shown in Figure 4 of the main text. [file 1743-8977-9-29-S3.pdf]

Mu Q et al

Additional File 3

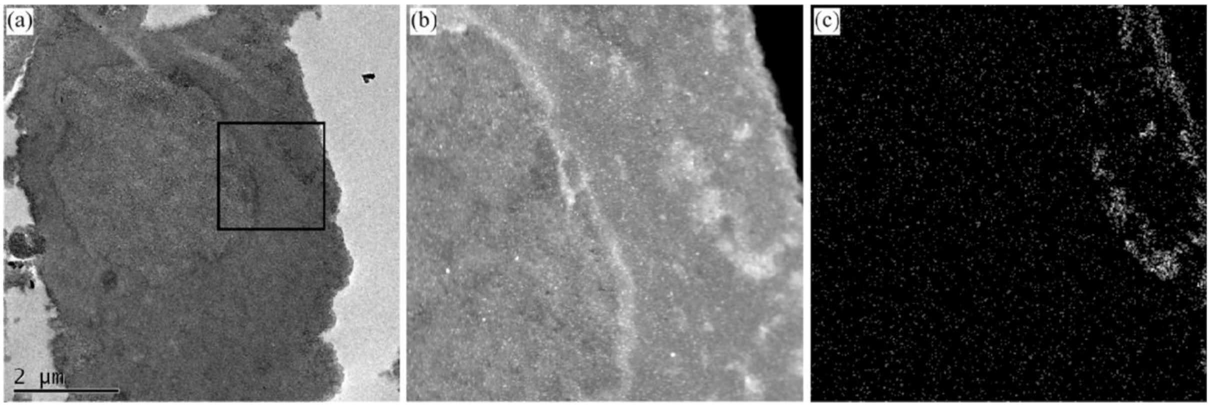

a) Bright field TEM image of A549 cell after 30 min incubation at 4°C with a 100 μg/ml of silica nanoparticles (Figure 4 of the main text). b) Dark field Scanning (S)TEM image of the boxed region in a). The bright contrast is due to strong scattering from the osmium tetroxide fixative decorating lipids and the silica nanoparticles. c) STEM map of the distribution of Si Kα X-rays from the imaged region in b). The bright contrast shows concentrated regions of silicon and this clearly demonstrates that the silica nanoparticles are localised to the cell membrane and that some have penetrated into the cytoplasm.
