# Supplementary material for: Mechanism of cellular uptake of genotoxic silica nanoparticles
Source: Part Fibre Toxicol. 2012 Jul 23;9:29. doi: 10.1186/1743-8977-9-29 (PMC3479067; doi:10.1186/1743-8977-9-29)
Supplement: Additional file 4 — Six figures showing TEM images of A549 cells after 30 min with and without 100 μg/ml silica NP exposures at 4°C and 37°C. [file 1743-8977-9-29-S4.pdf]

Mu Q et al

Additional File 4

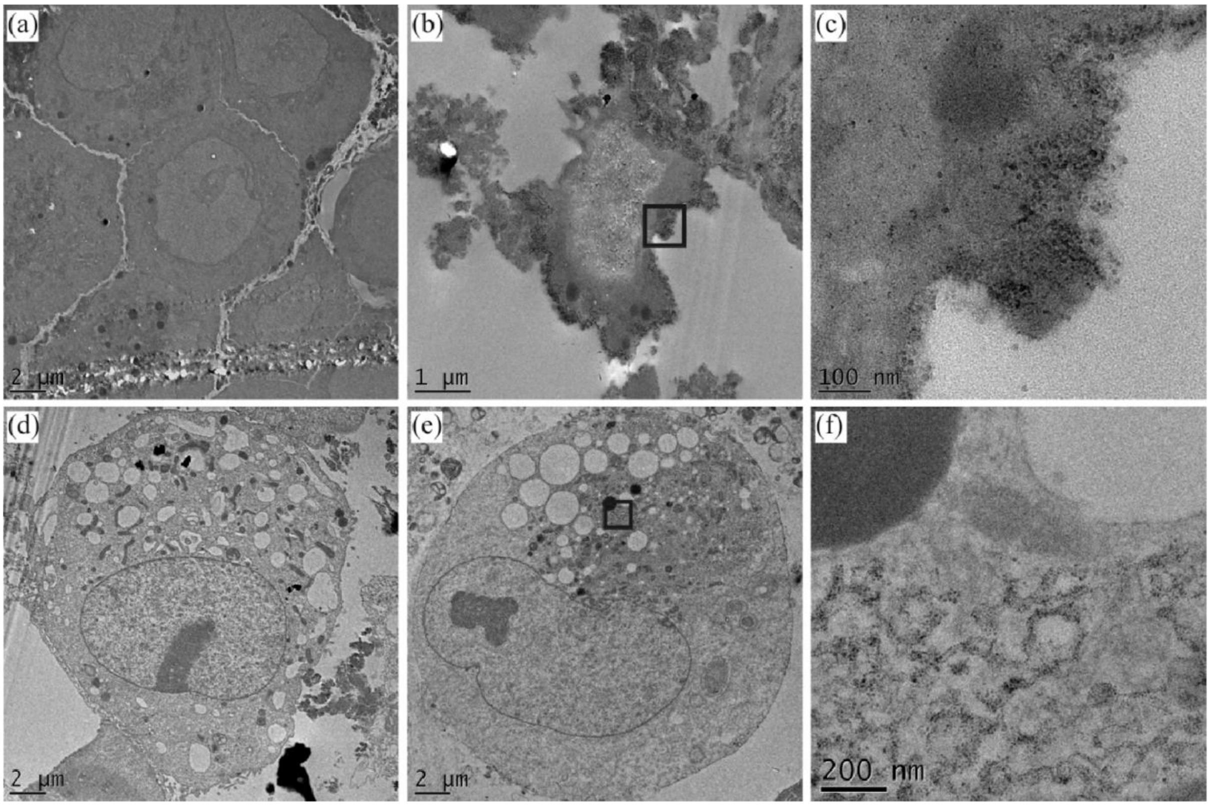

a) to c) Bright field TEM images of A549 cells incubated at 4 °C. a) Control cells.
The shrinkage and densification of the cells is likely to be a temperature effect. b) Cell after 30 min incubation with a 100 μg/ml of silica nanoparticles showing dense coverage of silica nanoparticles on the cell membrane. c) High magnification image of the boxed region in a), suggesting silica nanoparticle transmigration into the cell.
d) to f) Bright field TEM images of A549 cells incubated at 37 °C. a) Control cell. b) Cell after 30 min incubation with a 100 μg/ml of silica nanoparticles. c) |High magnification image of the boxed region in a) again demonstrating the presence of silica nanoparticles within the cell.
