# Supplementary material for: Mechanism of cellular uptake of genotoxic silica nanoparticles
Source: Part Fibre Toxicol. 2012 Jul 23;9:29. doi: 10.1186/1743-8977-9-29 (PMC3479067; doi:10.1186/1743-8977-9-29)
Supplement: Additional file 5 — Additional EM images of A549 cells incubated at 4°C for 30 min with a 100 μg/ml of silica NPs. [file 1743-8977-9-29-S5.pdf]

Mu Q et al

Additional File 5

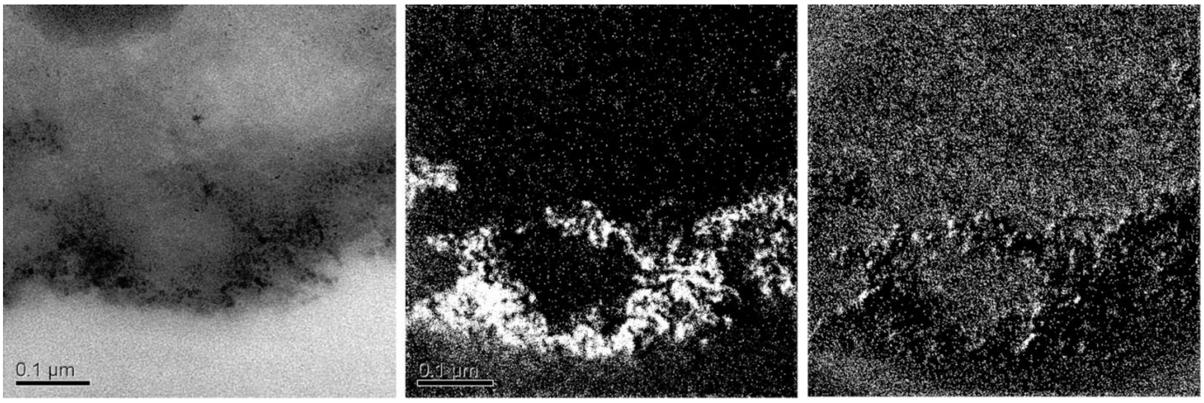

a) b) c)

a) Higher magnification bright field TEM image of A549 cells incubated at 4 ^{o}C for 30 min with a 100 µg/ml of silica nanoparticles .

b) Si $L_{2,3}$-edge, energy filtered (EF-) TEM elemental map confirming the penetration of the silica NPs into the cell alongside dispersion along the membrane (bright regions correspond to Si-rich areas)

c) P $L_{2,3}$-edge, EF-TEM elemental map distinguishing the cell from the resin support (again bright regions correspond to P-rich areas)

EF-TEM elemental maps were recorded using the standard three window technique described in Brydson, Electron Energy Loss Spectroscopy, BIOS: Oxford, (2001).
