# Supplementary material for: Mechanism of cellular uptake of genotoxic silica nanoparticles
Source: Part Fibre Toxicol. 2012 Jul 23;9:29. doi: 10.1186/1743-8977-9-29 (PMC3479067; doi:10.1186/1743-8977-9-29)
Supplement: Additional file 6 — Higher Magnification bright field TEM image and false colour elemental map of A549 cell incubated at 4°C for 30 min with a 100 μg/ml of silica nanoparticles. [file 1743-8977-9-29-S6.pdf]

Mu Q et al

Additional File 6

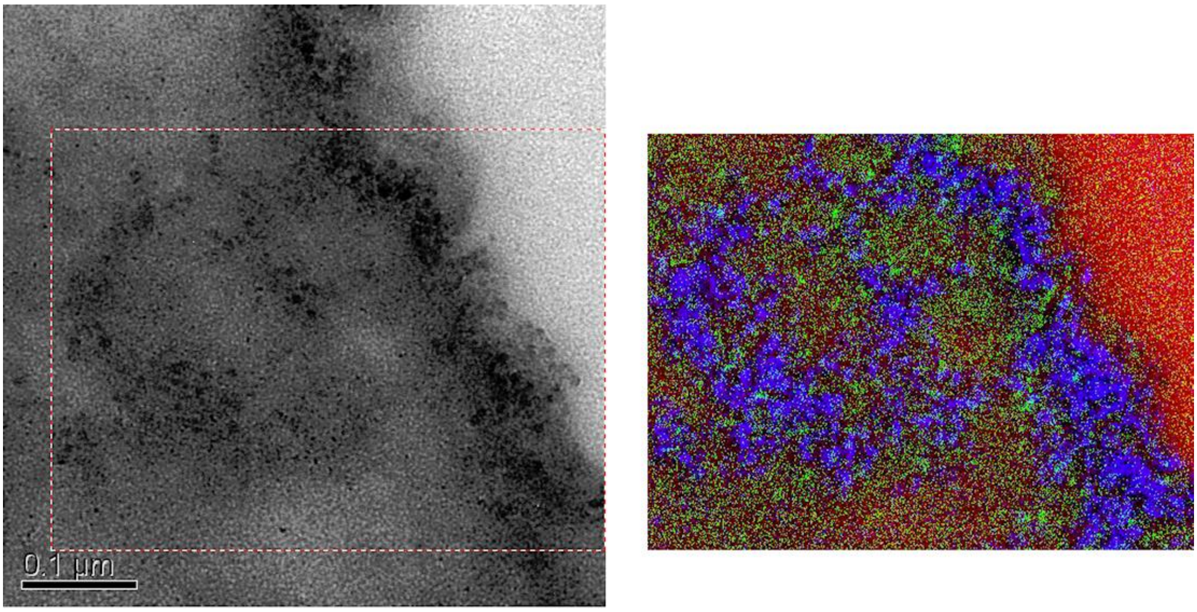

Red = BF Image Green = P map Blue = Si Map

a) Higher Magnification bright field TEM image of A549 cell incubated at 4 ^{o}C for 30 min with a 100 µg/ml of silica nanoparticles .

b) False colour composite map of boxed region in a), showing the relative distribution of Si (blue) and P (green) and again confirming the penetration of the silica NPs into the cell alongside dispersion along the membrane.

EF-TEM elemental maps were recorded as described in additional file 5 and then false coloured and overlaid to produce the image in b).
